# Implementation of an enhanced recovery program for complete cytoreductive surgery and hyperthermic intraperitoneal chemotherapy in a referral center: a case control prospective study

**DOI:** 10.1515/pp-2022-0133

**Published:** 2023-01-11

**Authors:** Diane Charleux-Muller, Thibaut Fabacher, Benoit Romain, Nicolas Meyer, Cécile Brigand, Jean-Baptiste Delhorme

**Affiliations:** Department of General and Digestive Surgery, Hautepierre Hospital, Strasbourg University Hospital, Strasbourg, France; Department of Public Health, Biostatistic laboratory, Strasbourg University Hospital, Strasbourg, France; INSERM Unit 1113, IRFAC, Strasbourg, France

**Keywords:** enhanced recovery after surgery (ERAS), enhanced recovery programs, hyperthermic intraperitoneal chemotherapy (HIPEC), peritoneal carcinomatosis

## Abstract

**Objectives:**

Current recommendations regarding enhanced recovery programs (ERPs) after complete cytoreductive surgery (CCRS) and hyperthermic intraperitoneal chemotherapy (HIPEC) are based on a low level of evidence. The aim of this study is to evaluate the effect of implementing an adapted ERP for CCRS and HIPEC in a referral center.

**Methods:**

We conducted a study with a prospective group of 44 patients (post-ERP group) who underwent CCRS with HIPEC between July 2016 and June 2018, the period during which ERP was implemented. This group was compared to a second retrospective group of 21 patients who underwent CCRS with HIPEC between June 2015 and June 2016, during which ERP was not yet implemented (pre-ERP group).

**Results:**

The ERP compliance rate was 65% in the post-ERP group. The hospital length of stay (HLS) was shorter in the post-ERP group: 24.9 days (IQR 11–68, pre-ERP group) vs. 16.1 days (IQR 6–45, post-ERP group), as was the major morbidity rate (pre-ERP group=33.3% vs. post-ERP group=20.5%). The nasogastric tube, urinary catheter and abdominal drains were all retrieved faster in the post-ERP group.

**Conclusions:**

The implementation of an adapted ERP after CCRS with HIPEC procedures reduces morbidity and shortens the HLS.

## Introduction

Enhanced recovery programs (ERP) for colorectal and upper gastrointestinal surgeries are effective in accelerating recovery and decreasing morbidity, hospital length of stay (HLS) and cost [1], [2], [3], [4], [5], [6]. In fact, ERP focuses on reducing surgical stress, encouraging rapid ambulation, and maintaining normal physiology in the postoperative period through changes made in the pre-, peri-, and post-operative periods. Today, these programs are highly recommended by the enhanced recovery after surgery (ERAS) society not only after colorectal surgery but also for other major abdominal surgeries, such as Ivor–Lewis esophagectomy and the Whipple procedure [1, 7], [8], [9].

Surgical management of primary or metastatic peritoneal malignancies consists of complete cytoreductive surgery (CCRS) with hyperthermic intraperitoneal chemotherapy (HIPEC) [10]. This approach improves the survival of patients with peritoneal pseudomyxoma, malignant peritoneal mesothelioma, and peritoneal metastasis from colorectal, ovarian, and gastric cancers [11], [12], [13], [14], [15], [16], [17].

For major surgeries, reducing morbidity associated with CCRS and HIPEC and alleviating the postoperative course on the patient have been primary concerns for expert teams. Over the past decades, the improved learning curve and increasing experience of surgical teams managing peritoneal neoplasms have led to a reduction in morbidity rate from 52 to 15% and in mortality rate from 10 to 0.9% [18].

With advances in ERAS, reports regarding its application in CCRS and HIPEC started appearing, with many showing promising results [3, 19], [20], [21], [22]. Furthermore, the ERAS society recently published guidelines concerning the perioperative management of CCRS and HIPEC for peritoneal diseases based on a standardized 2-round Delphi process of 24 experts [23, 24]. However, the level of evidence was low in 43% of cases, leading to weak recommendations in 49% of the guidelines [23, 24].

Because CCRS and HIPEC are major surgeries that usually last several hours and require specific pre-and postoperative management, all ERAS items may not be strictly applicable. The aim of this study is to propose an adapted ERP for CCRS and HIPEC in peritoneal diseases and evaluate the effect of its implementation in a referral center.

## Patients, materials and methods

In June 2016, an ERP adapted from the ERAS Society guidelines for colorectal surgery was introduced and implemented for all patients with peritoneal diseases presenting for a scheduled CCRS and HIPEC in the Department of Digestive Surgery at Hautepierre Hospital, Strasbourg, France. Between June 2016 and June 2018, 44 patients were included (post-ERP group), and data were prospectively recorded on a daily basis by a dedicated nurse and included data in the preoperative, intraoperative, and postoperative periods. Those were compared to a control group of 21 patients who presented for the same procedures between June 2015 and June 2016 before the ERP was implemented (pre-ERP group), and data were retrospectively collected. In our institution, CCRS and HIPEC have been performed since 2005; thus, this close comparative period was chosen to limit biases related to time and surgeon experience.

Informed consent was obtained from all patients included in this study. The study complies with the World Medical Association’s Helsinki statement regarding the ethical conduct of research involving human subjects.

### Surgical procedure

Surgical indications were discussed and validated in multidisciplinary meetings. A median xyphopubic incision was systematically used, and the abdominal cavity was completely explored. The extent of peritoneal seeding was calculated for each patient using Sugarbaker’s peritoneal cancer index (PCI) [10, 25, 26]. CCRS consisted of the excision of all tumoral deposits as described by the peritonectomy procedure [10]. At the end of CCRS, cytoreduction was evaluated using the CC score [10]. In our center, if CC0 was judged unachievable, HIPEC was contraindicated. In cases where completed resection of liver metastasis was feasible, radiofrequency ablation or minor hepatectomy (defined by less than three contiguous liver segments according to the Couinaud definition) was ensued [27]. Following CC0 CCRS, HIPEC was performed in a closed abdominal cavity at 42 °C with abdominal massage. Chemotherapy agents were chosen according to the disease.

For mitomycin C (MMC)-based HIPEC, MMC was administered intraperitoneally at a dose of 0.8 mg/kg in a peritoneal dialysis solution for 90 min. For oxaliplatin-based HIPEC, patients received an intravenous perfusion of 5-FU (400 mg/m^2^) with leucovorin (20 mg/m^2^) immediately before starting HIPEC. Oxaliplatin was then administered at a dose of 360 mg/m^2^ in an iso-osmotic 5% dextrose solution for 30 min. Cisplatin was either used alone at a dose of 75 mg/m^2^ or jointly administered with MMC at doses of 0.8 mg/kg and 0.5 mg/kg, respectively, or with doxorubicin at doses of 50 mg/m^2^ and 15 mg/m^2^, respectively, in a peritoneal dialysis solution. Because we performed a closed skin technique, we systematically reopened the abdominal cavity, and abdominal drains were placed before final closure. In the event where diaphragmatic peritonectomy was performed, bilateral chest tubes were also placed.

### Enhanced recovery program (ERP)

The ERAS colorectal society guidelines entail the application of 25 items [2]. Our adapted ERP contained 16 items (Table 1), of which 6 were adapted for CCRS and HIPEC as seen clinically relevant. Hypothermia management was not included, as we used an upper body forced air heating system routinely in CCRS even before ERP implementation. Prevention of nausea and vomiting and prevention of postoperative ileus were merged under “Prevention of postoperative nausea, vomiting and ileus” (PPNVN). The prehabilitation consisted of an immunonutrition administered orally one week before the surgery as well as encouraging physical activity and pulmonary physiotherapy. Bowel preparation was routinely administered for all patients and consisted of 3 L of oral polyethylene glycol (PEG) solution without antibiotics the day before the surgery [28, 29]. The systematic audit of our practice is performed by this study to allow comparison with other institutions and recently published guidelines [22, 30].

**Table 1:** Description of the compliance to the ERP criteria.

ERP items	Applied criteria	Pre-ERP group (n=21) (n), %^a^	Post-ERP group (n=44) (n), %^a^
1. ERP information	Specific to the program, oral and written preadmission information	(21) 0%	(37) 84.1%
2. Preoperative bowel preparation	Routine oral bowel preparation	(19) 90.5%	(31) 70.4%
3. Prehabilitation	Immunonutrition	(20) 95.2%	(43) 97.7%
4. Fasting liquid respect	Sweet drink 2 h before surgery, and 6 h for solids	(19) 90.5%	(29) 65.9%
5. Preanesthetic medication	No medications to cause long-term sedation	0.00%	(30) 68.2%
6. Thromboprophylaxis	Subcutaneous low-dose unfractionated heparin or subcutaneous low-molecular-weight heparin	(21) 100%	(32) 100%
7. Antimicrobial prophylaxis delay	Between 25 min and 1 h 15 min before incision	(14) 66.6%	(26) 81.2%
8. Perioperative fluid management	<2,500 mL	(3) 14%	0.00%
9. Postoperative nausea, vomiting, and ileus prevention (PPNVN)	Antiemetic, AINS, steroids, at least one of the three	(14) 66.6%	(36) 81.8%
10. Nasogastric tube removal	If removed before POD 4^b^	(6) 28.6%	(34) 77.3%
11. Postoperative fluid management	≤2,000 mL at POD 3	(2) 9.5%	(1) 2.3%
12. Standard anesthetic protocol and epidural catheter	Avoid long-acting opioids, midthoracic epidural recommended preoperatively	(19) 90.5%	(42) 95.4%
13. Urinary catheter removal	Before POD 5^b^	(6) 28.6%	(20) 45.4%
14. Pain management	If EVA<4 at POD3	(16) 76.2%	(21) 47.7%
15. Solid food	If present at POD 4^b^	(2) 9.5%	(12) 27.3%
16. Early mobilization	If walking at POD 2^b^	(5) 22%	(32) 100%
ERP score		55.5%	65.3%

^a^Percentage considered in the total analysis. ^b^Adapted from our initial ERP for CCRS and HIPEC. POD, postoperative day; ERP, enhance recovery program; Min, minute.

### Adaptation of the ERP items to CCRS and HIPEC

Based on previously published series at the time of implementation of the ERP, four items from the ERAS society guidelines for colorectal surgery were adapted for CCRS and HIPEC [19, 20]. Nasogastric tube removal was changed from postoperative day (POD) 0 to POD 4. Urinary catheter removal was changed from POD 0 to POD 5. Ambulation was changed from POD 0 to POD 2. Normal diet administration was switched from POD 1 to POD 4.

We then compared the application of the ERP criteria and their compliance according to the two periods (pre-ERP vs. post-ERP periods). The ERP score was defined as the total compliance rate to the ERP and corresponded to each criterion (defined in Table 1) for each patient (Yes=1, No=0), which provided a score between 0 and 100. The ERP score was obtained by the addition of all the scores divided by the total number of items, which was 16.

### Postoperative morbidity

Postoperative mortality and morbidity were evaluated according to the Dindo–Clavien classification within 90 days after surgery [31]. Complications of grade III or higher were considered major morbidities.

### Statistical analysis

For descriptive analysis, we used medians and standard deviations (SDs) for continuous variables and frequencies and percentages for categorical variables. We used Bayesian inference to compare the ERP criteria according to the two periods and to the percentages of application. We computed the probability that the between-group difference was larger than 0 (Pr (diff>0)). Bayesian methods do not use p values, and the computed probabilities should not be confused with p values. Probabilities near 1 or 0 suggest an effect, respectively, of a positive or negative difference.

For each criterion, we chose a little informative beta law and entered it on the overall mean of the whole population. To compare the total mean ERP score between the two groups, we used prior information from a noninformative gamma law. Throughout our entire cohort, we searched for predictive factors that can improve major postoperative morbidities (according to the Clavien‒Dindo grade III to V). A statistical Bayesian analysis was preferred due to the small cohort size. Exact results are given, even for small sample sizes, contrary to other methods, which usually rely on asymptotic results and approximation, resulting in more informative conclusions than simple p values. Analysis was performed with R 3.5, jags and the GMRC shiny Stats software based on R [32], [33], [34].

## Results

### Patient characteristics

A total of 65 patients were included: 21 in the pre-ERP group and 44 in the post-ERP group. The median age was 65 years (44–76 years) in the pre-ERP group and 56 years (20–76 years) in the post-ERP group. The population was more likely to be female in the pre-ERP group (85.7%) than in the post-ERP group (59.1%). The description of the ERP and its compliance rates are described in Table 1.

Patient characteristics are summarized in Table 2. Peritoneal disease was most common in the colon (71.4% in the pre-ERP group vs. 55.8% in the post-ERP group). Other etiologies included the stomach (4.8% in the pre-ERP group vs. 16.3% in the post-ERP group), the appendix (14.3% in the pre-ERP group vs. 4.6% in the post-ERP group), the ovary (4.8% in the pre-ERP group vs. 11.6% in the post-ERP group), the small bowel (0% in the pre-ERP group vs. 4.6% in the post-ERP group) and primary peritoneal neoplasms (4.8% in the pre-ERP group vs. 4.6% in the post-ERP group).

**Table 2:** Patient characteristics.

Characteristics	Pre-ERP group (n=21)	Post-ERP group (n=44)
Gender (female), n (%)	18 (85.7)	26 (59.1)

Age, mean, (SD)	64.8 (7.46)	56.0 (13.3)

BMI, mean (SD)	24.7 (4.9)	25.5 (4.2)
ASA^a^ 1, n (%)	0	8 (18.2)
ASA^a^ 2, n (%)	11 (64.7)	21 (47.7)
ASA^a^ 3, n (%)	6 (35.3)	14 (31.8)
ASA^a^ 4, n (%)	0	1 (2.27)
WHO^b^ performance status 0, n (%)	15 (100)	37 (84.1)
WHO^b^ performaces status 1, n (%)	0	5 (11.4)
WHO^b^ performance status 2, n (%)	0	2 (4.5)

Surgical procedures, n (%)		

Radical omentectomy	41 (93.2)	32 (100)
Diaphragmatic peritonectomy right	6 (28.6)	13 (29.5)
Diaphragmatic peritonectomy left	2 (9.5)	6 (13.6)
Pelviperitonectomy	14 (66.7)	26 (59.1)
Bowel resection	13 (61.9)	25 (56.8)

Number of digestive anastomosis, n (%)		

0	8 (38.1)	16 (37.2)
1	12 (57.1)	13 (30.2)
2	0	11 (25.6)
3	1 (4.8)	3 (7.0)

Perioperative transfusion, mean, (SD)		

Platelet units	49 (5.5)	2.7 (6.0)
Packed red blood cell	6.3 (9.6)	4.2 (4.8)

HIPEC regimen, n (%)		

Cisplatin	2 (9.5)	4 (9.1)
Cisplatin and doxorubicin	0	1 (2.3)
Mitomycin C	10 (47.6)	19 (43.2)
Mitomycin C and cisplatin	0	3 (6.8)
Oxaliplatin	9 (42.9)	17 (38.6)

PCI, mean, (SD)	9.8 (8.7)	8.4 (7.5)

Primary tumor site, n (%)		

Appendix	3 (14.3)	2 (4.6)
Colon	15 (71.3)	24 (55.9)
Stomach	1 (4.8)	7 (16.4)
Small bowel	0	2 (4.6)
Ovary	1 (4.8)	6 (13.9)
Pseudomyxoma	1 (4.8)	2 (4.6)

^a^ASA, American Society of Anesthesiologists; ^b^WHO, World Health Organization.

A protective stoma was performed in 27.5% of the cases in the pre-ERP group vs. 23.8% in the post-ERP group. The mean operative time was 524 min (SD=118) in the pre-ERP group vs. 499 min (SD=122) in the post-ERP group. The mean PCI was 9.8 (SD=8.7) in the pre-ERP group and 8.4 (SD=7.5) in the post-ERP group.

### ERP compliance

The compliance rate to the ERP was 55.5% in the pre-ERP group and 65.3% in the post-ERP group, as described in Table 1.

The adherence to the ERP was improved between the two groups in the following items: prehabilitation, delay in prophylactic antibiotic administration, PPNVN, pain management by epidural thoracic catheter, urinary catheter removal, and resumption of solid food intake. According to our analysis, ERP compliance (measured by the ERP score) is 95.3% better in the post-ERP phase than in the pre-ERP phase.

### ERP outcomes and morbidity

Intensive care unit stay (ICU) and the HLS were decreased in the post-ERP group (Table 3). The nasogastric tube, urinary catheter and abdominal drains were all removed faster in the post-ERP group (Table 3). However, 11 patients (25%) in the post-ERP group required a new nasogastric tube insertion after early removal.

**Table 3:** Postoperative characteristics.

	Pre-ERP group (n=21)	Post-ERP group (n=44)
Mean ICU stay (days) mean, (SD)	12.4 (7.3)	9.3 (6.5)
Mean hospital stay (days) mean, (SD)	24.9 (15.1)	16.1 (8.3)

Postoperative management		

Urinary catheter POD removal, mean, (SD)	7.3 (3.5)	6.4 (3.1)
Drainage POD removal, mean, (SD)	10.7 (3.8)	8.4 (2.9)
Epidural catheter POD removal of the average mean, (SD)	4.4 (1.8)	4.7 (1.9)
Opioid patient-controlled analgesia POD removal, mean, (SD)	5.4 (2.0)	6.0 (3.5)
Resumption transit delay, mean, (SD)	4.9 (2.5)	3.1 (1.5)
Nasogastric tube POD removal, mean, (SD)	6.2 (3.7)	3.5 (3.0)
Nasogastric tube replacement, n (%)	0 (0)	11 (25)

POD, postoperative day; ICU, intensive care unit; SD, standard deviation; N, number.

Twenty-six percent of all patients had a major complication (Dindo–Clavien≥III). The overall major morbidity rate was lower in the post-ERP group: seven patients (33.3%) in the pre-ERP group vs. 10 patients (22.7%) in the post-ERP group. One postoperative death occurred in the post-ERP group due to acute myocardial ischemia. Bleeding was also observed (14.3% in the pre-ERP group and 6.8% in the post-ERP group), likely related to oxaliplatin-based HIPEC. Mesenteric ischemia and anastomotic leakage only occurred in the post-ERP group (11.3%). Infectious complications occurred in 4.7 and 2.3% of patients in the pre-ERP and post-ERP groups, respectively. One hemorrhagic stroke and 2 cases of respiratory distress were also reported in the pre-ERP group.

The female population seems to have more major morbidity (n=14 (87.5%)) than the male population (n=2 (12.5%)). Patients with major morbidity had a higher mean PCI (mean=11.6; SD=8.15) than patients with minor morbidity (mean=7.8; SD=7.54). A higher level of respiratory capacity by spirometry was observed between the group with major morbidity (mean 435, SD 147) compared to those with minor morbidity (mean 288, SD 93). Criteria such as immunonutrition, physical activity, primary tumor site, number of digestive resections, number of peritonectomies, initial comorbidities, and BMI are similar depending on the type of complication.

Patients who adhered to the eight following items had a 71.2% higher probability of having decreased major morbidity than those who did not: ERP information, anesthetic premedication, perioperative fluid management, nasogastric tube removal at POD 4, standard anesthetic protocol and epidural catheter, efficient pain management, urinary catheter removal at POD 5, and early mobilization at POD 2. The probability that the percentage of major morbidity in the pre-ERP phase was higher than the percentage in the post-ERP phase was 83%.

## Discussion

The implementation of an ERP is a gradual and progressive road, with different steps to achieve. We started fully adopting this modern aspect of perioperative management in 2015 and have continued to progress toward a well-tailored ERP. Many surgeons performing CCRS and HIPEC have undergone the same path by progressively extrapolating ERAS practices from colorectal and gynecological guidelines [35]. Many of the benefits of an ERP come from standardizing perioperative management more than from the individual effect of each component of the ERP [36].

The major morbidity rate reported in our study (22.7%) is comparable to the most recent literature [37, 38].

The ERP protocol has been shown to be effective in several abdominal surgeries [39] and in cancer-related mortality for colorectal cancers [40]. We truly believe this can be adapted for CCRS with HIPEC with the same goal of improving the postoperative course for those patients.

Other papers have previously emphasized the difficulty of applying an ERP in CCRS and HIPEC [21, 22, 30, 41]. In 2011, Cascales-Campos et al. showed that the introduction of a multimodal rehabilitation program was reasonable in a series of 46 selected patients with low volume carcinomatosis (PCI=12.35, IQR: 3–21) after CCRS with HIPEC for peritoneal carcinomatosis of ovarian origin. In 2016, the same team prospectively analyzed 156 patients after CCRS with HIPEC in which an ERP was implemented. As in our study, 16 of the 20 items recommended by the ERAS Society were included in their study, but their protocol was limited to patients who had no more than one digestive anastomosis, unlike our study, where 32.6% of patients had more than one digestive anastomosis. In their series, only 3.8% needed reinsertion of a nasogastric tube for paralytic ileus, vs. 25% in our case-control cohort. This finding can be explained not only by our higher rate of digestive anastomoses and the administration of oral bowel preparation but also by the mismanagement of perioperative fluid administration [22]. A retrospective review by Siddharthan et al. compared 16 and 15 patients who underwent CCRS with HIPEC before and after the implementation of an ERP protocol between 2015 and 2018 in a single institution [30]. The study reports the feasibility of an ERP in patients undergoing CCRS with HIPEC with improved HLS (11 days before ERP vs. 7 days after ERP) without increased morbidity or mortality. Webb et al. also reported a decrease in HLS from 10.3 ± 8.9 days to 6.9 ± 5.0 days (p=0.007) and in the major complication rate from 24 to 15% (p=0.243) following ERP implementation. A recent feasibility study including 31 patients reported a decrease in HLS (9–6 days p=0.002), without a significant difference in the 30-day readmission rate or complication rate [41]. Hospitalization in intensive care corresponds in our hospital to a unit where the monitoring and the number of nurses is slightly higher than a conventional service. The level of care (drugs, implementation of invasive procedures) is, however, identical to the conventional service. ICU hospitalization should be considered in our study as an enhanced conventional service, which is likely the reason why hospitalization in the ICU seems longer compared to the literature.

The ERP may also help reduce opioid use during and after hospitalization (ERP total morphine equivalents 156 vs. non-ERP of 856, p<0.001 during hospitalization; ERP 55% of patients, non-ERP 97%, p<0.02 after hospitalization) [42]. The main study on the matter was published by White et al. and included 168 patients: 88 in the pre-ERP group and 80 in the ERP group. The ERP group received less intraoperative fluids (mean, 4.2 vs. 6.4 L; p<0.01), the mean LOS was 7.9 days post-ERP compared with 10.0 days pre-ERP (p=0.015), and Clavien‒Dindo complications grade≥3 were lower after ERP (23.7 vs. 38.6%; p=0.04) [43].

Finally, a recent review of the impact of ERP, including six retrospective studies, showed that implementing an ERP after CCRS with HIPEC is feasible and may decrease the HLS (MD: −2.82 95% CI: −3.79, −1.85 I2=29% p<0.00001) as well as the major morbidity rates of Clavien‒Dindo III/IV (RR: 0.60 95% CI: 0.41, 0.87 I2=0% p=0.007) compared to the control group [38]. However, the findings only suggest that ERP after CCRS with HIPEC is not associated with increased readmission, reoperation, or mortality rates.

A recent Indian online survey was performed to study clinicians’ practice about ERP in patients undergoing CCRS with HIPEC: the adoption of postoperative practices was lower than other intraoperative practices. They thus propose a prospective study to create an adapted protocol [35].

The ERAS Society proposed guidelines after CCRS with HIPEC: the recommendations were strong for preoperative and intraoperative management, specifically concerning anesthesia and pain management, preoperative nutrition, carbohydrate loading, perioperative glucose control and immunonutrition. However, the postoperative management guidelines were based on a low level of evidence, leading to weak recommendations with a low possibility of extrapolations and imprecise data. As emphasized by the ERAS Society guidelines, there is an urgent need to further investigate the different aspects of perioperative care for CRS ± HIPEC to generate more and better primary evidence [23, 24].

This study can fulfill this need. Based on our results and experience, we were able to propose the following adapted postoperative ERP:

– Early ambulation on POD 1
– Optimal pain management with epidural catheter until POD 4
– Nasogastric tube removal before POD 4, with continued nausea preventive treatment by steroids and prokinetics
– Oral solid food intake starting on POD 4
– Urinary catheter removal before POD 5
– Postoperative fluid restriction to ≤2 L/day

Although the overall compliance rate was improved by only 10%, the adjustment of criteria to CCRS and HIPEC may have contributed to decreasing postoperative morbidity and HLS.

Visceral edema, which is highly induced by excessive intravenous perfusion, is one of the main ileus-related factors. Thus, overhydration should be discussed according to the chemotherapy drug used in HIPEC. The use of cisplatin requires intravenous overhydration to prevent acute kidney injury, but mainly during the first two postoperative days. For other chemo-based HIPEC, intravenous hydration should be reduced to the absolute minimum. The difficult postoperative management of these patients requires an interdisciplinary approach involving anesthesiologists, surgeons, and nurses.

Our study reports the largest French cohort, which compares both periods before and after implementing an ERP for patients who underwent a CCRS with HIPEC. This study proposes several protocoled items to follow in daily practice. One of the strengths of our study is the absence of patient selection according to their expected postoperative morbidity. Despite the relatively short period of data collection allowing standardized and homogenous patient management, the decrease in the use of oxaliplatin as well as the decrease in the PCI between the two periods may somewhat explain the decrease in morbidity. The female population seems to have more serious complications, but notably, the pre-ERP phase had more females. Historical group comparisons may introduce some surgical experience and technical bias on the outcomes; however, because the pre-ERP group analysis started after a 10-year experience in CCRS and HIPEC, those effects are limited.

We acknowledge some limitations in our study, such as the modest number of patients and its single-center design. One should note that although this study does not show direct connections, the correlation with ERP and its positive outcomes are clearly demonstrated. Based on the current ERAS post CRS and HIPEC recommendations, we should introduce new elements of postoperative rehabilitation to implement in future studies: postoperative control of glucose, postoperative analgesia with thoracic epidural analgesia, postdischarge care (nutritional care and physiotherapy), and prevention, early detection, and treatment of HIPC complications.

## Conclusions

Improving the ERP compliance rate by 10% led to a decrease in the morbidity rate from 33.3% to 22.7% and in the HLS by 6 days. We postulate that the higher the compliance rate is, the better the results will be. Nevertheless, some items of the ERP must be adapted to heavy surgeries. The aim of this pilot study was to propose a clinical basis for future ERP implementations that need to be confirmed by prospective, multicentric studies serving as a complement to ERAS society guidelines. This study may reinforce the strength of ERAS society recommendations and can be a useful tool for physicians involved in peritoneal neoplasm surgeries.

## Highlights

– Surgical management of primary or metastatic peritoneal malignancies consists of complete cytoreductive surgery (CCRS) with hyperthermic intraperitoneal chemotherapy (HIPEC).
– Enhanced recovery programs (ERP) after colorectal and upper gastrointestinal procedures are effective in reducing morbidity and accelerating recovery after surgery.
– Reducing CCRS and HIPEC morbidity is a primary concern for the surgeon and for his team.
– The implementation of an adapted ERP for CCRS and HIPEC may reinforces the recent ERAS society recommendations.
